# Changing Trends in the Epidemiology and Risk Factors of *Pneumocystis* Pneumonia in Spain

**DOI:** 10.3389/fpubh.2019.00275

**Published:** 2019-10-04

**Authors:** Estefanía Pereira-Díaz, Fidel Moreno-Verdejo, Carmen de la Horra, José A. Guerrero, Enrique J. Calderón, Francisco J. Medrano

**Affiliations:** ^1^Internal Medicine Service, Hospital Universitario Virgen del Rocío, Seville, Spain; ^2^Area of Cardiovascular and Respiratory Diseases, Instituto de Biomedicina de Sevilla (IBiS)/CSIC, Seville, Spain; ^3^Centro de Investigación Biomédica en Red de Epidemiología y Salud Pública, Seville, Spain; ^4^Clinical Documentation Service, Hospital Universitario Virgen del Rocío, Seville, Spain; ^5^Department of Medicine, Universidad de Sevilla, Seville, Spain

**Keywords:** pneumonia, *Pneumocystis*, epidemiology, HIV infections, Spain

## Abstract

**Objective:** The information about the epidemiology of *Pneumocystis* pneumonia (PcP) in Europe is scarce, and in Spain there are only data nationwide on patients with HIV infection. This study has been carried out with the aim of knowing in our country the current epidemiological spectrum and the risk factors of PcP.

**Methods:** Observational, descriptive transversal study that included all patients admitted in Spain with diagnosis upon discharge of PcP registered in the National Health System's Hospital Discharge Records Database of Spain, between 2008 and 2012.

**Results:** Four thousand five hundred and fifty four cases of PcP were reported, 1,204 (26.4%) in HIV-negative patients. During the study period, mean annual incidence (cases per million) was 19.4, remaining globally stable, increasing from 4.4 to 6.3 in HIV-negative patients and decreasing from 15.5 to 13.4 among HIV-infected patients. Risk factors were identified in 85.5% of HIV-negative cases, the most frequent being hematological neoplams (29%), chronic lung diseases (15.9%), and non-hematological cancers (14.9%). Mean mortality and hospitalization cost were high (25.5% and 12,000 euros, respectively).

**Conclusions:** The results of this first nationwide study in Spain allow a change in the misconception that, after the AIDS epidemic, PcP is an infrequent disease, showing that today it is an emerging problem in patients without HIV infection. These findings underlines the need for increased efforts toward a better characterization of risk groups to improve prophylactic strategies and reduce the burden of disease.

## Introduction

*Pneumocytis jirovecii* continues to be one of the major opportunistic pathogens that affect individuals with acquired immune deficiency syndrome (AIDS) and patients with immunosuppression due to other causes, in which it causes severe pneumonia with high morbi-mortality (1–3). Until 1980, it was an uncommon disease that affected malnourished children with severe immunodeficiencies and adults with situations of intense immunosuppression, mainly associated with chemotherapy in cancer. With the emergence of the AIDS pandemic, the prevalence of *Pneumocystis* pneumonia (PcP) increased drastically, and it became the most common AIDS-defining disease in developed countries (1, 3).

After the introduction of chemoprophylaxis with co-trimoxazole in HIV-infected patients with a CD4^+^ lymphocyte count lower than 200 cells/mm^3^ from 1989 and, above all, after the generalization of highly active antiretroviral therapy (HAART) in the mid-'90s, an important decrease in the incidence of PcP was observed in developed countries (3), which in Europe fell from 4.9 cases per 100 persons-year before 1995 to 0.3 cases per 100 persons-year after 1998 (4). Despite this, PcP continues to be a non-infrequent disease in patients with HIV infection, both in developed countries and in some areas of the third world in which HIV is endemic, where access to chemoprophylaxis and antiretroviral drugs is limited (1, 5).

Additionally, an increasing number of PcP cases are currently being described in immunosuppressed patients without HIV infection (1, 2), due to the increased use of immunosuppressive drugs and chemotherapy in people with cancer, bone marrow, or solid organ transplants and autoimmune diseases in developed countries (2, 6–8).

Specific chemoprophylaxis with trimethoprim-sulfamethoxazole, dapsone, or atovaquone is effective for preventing PcP in patients with HIV infection and also in subjects without infection (6, 7, 9), although in the latter group the circumstances associated with a greater risk of symptomatic infection are poorly defined. As such, to reduce the incidence of this disease in patients not infected with HIV, it is necessary to identify the high-risk groups, which should receive prophylaxis and/or close monitoring in order to be able to carry out an early diagnosis of the disease.

Moreover, the frequency of PcP seems to be increasing in some patient subgroups without HIV infection, such as those who have received a kidney transplant (10). These findings suggest that the clinical-epidemiological characteristics of PcP could currently be changing. However, the information about the epidemiology nationwide on the current situation of PcP in Europe is scarce (11), and in Spain there are only data on patients with HIV infection (12). Therefore, this study has been carried out with the aim of knowing in our country the current epidemiological spectrum and the risk factors of PcP.

## Materials and Methods

### Design

Observational, descriptive transversal study that included patients admitted in Spain with diagnosis upon discharge of PcP registered as code 136.3 of the International Classification of Diseases, Ninth Revision, Clinical Modification (ICD-9-CM), listed in any position in the Hospitalization Minimum Data Set (CMBD), that is the National database of hospital discharge records in Spain, between 2008 and 2012. The project was approved by the Clinical Research Ethics Committee (CEIC) of the Hospital Universitario Virgen del Rocío.

### Variables

First at all, an exploratory analysis of the database was carried out to identify possible outliers, duplicates, and lost values. When the same patient had several hospitalization episodes, only the variables of the first episode were included in the analysis. There was no gaps in the dataset (each calendar year was fully covered).

The data collected in the CMBD database were the discharge year, the admission date, sex, age, HIV infection diagnosis, weight of the “All Patient Refined Diagnosis-Related Groups” (APR-DGRs), cost of the process in euros, days hospitalized, death during hospitalization and re-admission within 30 days after discharge. When the same patient had several hospitalization episodes during different calendar periods, readmissions were counted only once. The annual incidence rate of PcP (per million inhabitants) was calculated considering the data of the Spanish Statistical Office (INE) of Spain (13) for the study years.

Moreover, the study researchers identified a risk category in patients without HIV infection. For the coding of this variable, the tool validated for clinical studies “Clinical Classifications Software (CCS) for ICD-9-CM” (14) was used. The CCS variables were grouped into nine mutually exclusive categories that, frequently, were as conditions or diseases associated with PCP (2, 6, 8, 9, 11, 15, 16). If there were two or more risk categories in the same patient, considering the data on incidence and/or frequency of PcP reported in the literature (8, 11, 16) the following order of preference for recording the risk category was followed: (1) active with chemotherapy, (2) hematologic malignances, (3) malignances other than hematologic, (4) any transplant, (5) autoimmune diseases, including rheumatoid arthritis, systemic lupus erythematosus, polymyositis, dermatomyositis, chronic mixed connective tissue disease, Crohn's disease, and systemic vasculitis, (6) chronic lung diseases, (7) chronic nephropathies, (8) hematologic disorders other than malignances, (9) chronic liver diseases, (10) unknown risk factor.

Two study researchers (EP and FM) reviewed all of the records independently and assigned each of the cases to one of these risk categories. The discrepancies in the categorization (which affected <5% of patients) were resolved by a third researcher (EC).

### Statistical Analyses

Results are expressed as mean values ± standard deviation (SD). The chi-square test was used for assessing differences between proportions. For continuous variables, levels of significance were calculated with the one-way analysis of variance (ANOVA) test for parametric variables and with the Mann-Whitney *U*-test and the Kruskal-Wallis *H*-test for non-parametric variables. Temporal trends in the incidence of PcP were investigated by Poisson regression. The results were considered statistically significant at *p* < 0.005. Statistical analyses were performed by using the Statistical Package for Serial Studies for personal computers (IBM SPSS version 22.0, IBM Corporation, Somers, NY, USA).

## Results

### PcP Incidence

During the 2008–2012 period, CMBD recorded a total of 4,554 cases of PcP. Of these, 1,204 (26.44%) were recorded in patients without HIV infection. Overall incidence of PcP remained stable during the observation period (mean annual rate for the period of 19.4, coefficient –0.007, *p* = 0.47). The incidence rate in patients without HIV infection increased significantly from 4.4 per million in 2008 (*n* = 201 cases) to 6.3 in 2012 (*n* = 299 cases) (coefficient 0.08, *p* < 0.001), and amongst those infected by HIV decreased from 15.5 to 13.4 per million (coefficient −0.03, *p* = 0.001) (Table 1).

**Table 1:** Annual incidence rate of *Pneumocystis* pneumonia (PcP), Spain, 2008–2012.

**Year**	Spanish population^*^	**Total**		**HIV-negative**		**HIV-positive**	
		**PcP cases**	**Incidence rate§ (95% CI)**	**PcP cases**	**Incidence rate§ (95% CI)**	**PcP cases**	**Incidence rate§ (95% CI)**
2008	46,157,822	917	19.9 (18.6–21.2)	201	4.4 (3.8–5)	716	15.5 (14.4–16.7)
2009	46,745,807	986	21.1 (19.8–22.4)	256	5.5 (4.8–6.2)	730	15.6 (14.5–16.8)
2010	47,021,031	833	17.7 (16.5–19)	192	4.1 (3.5–4.7)	641	13.6 (12.6–14.7)
2011	47,190,493	884	18.7 (18–20)	256	5.4 (4.8–6.1)	628	13.3 (12.3–14.4)
2012	47,265,321	934	19.8 (19–21.1)	299	6.3 (5.6–7.01)	635	13.4 (12.4–15)
*p* (coefficient)^**^			0.476 (−0.007)		<0.001 (0.08)		0.001 (−0.03)

* *Source: Instituto Nacional de Estadística (Spain); § per million inhabitants; CI, (confidence interval)*;

** * Temporal trends were investigated by Poisson regression. A positive coefficient indicate an increase in the incidence and a negative value a decrease in the incidence*.

### Demographic Characteristics, Clinical Evolution, and Consumption of Resources

Compared to patients with HIV infection, patients without HIV infection had a higher mean age (58 vs. 42.1 years of age) and the proportion of men was higher (74.1 vs. 60.5%). Hospital stay (24.9 vs. 22 days), the DRG weight (2.8 vs. 2.7) and the cost of hospitalization (12,137 vs. 11,436 euros) were also higher and they had a worse clinical evolution, with higher rates of intrahospital mortality (25.5 vs. 13.6%) and re-admissions (24.9 vs. 10.8%) (Table 2). The annual evolution of the different variables in the study period in patients without HIV infection is displayed in Table 3. There were only statistically significant differences for the age variable (*p* = 0.002), the sex (*p* = 0.022) and the cost per episode of hospitalization (*p* = 0.001).

**Table 2:** Epidemiologic, hospitalization cost, and outcome of patients with *Pneumocystis* pneumonia, Spain, 2008–2012.

	**Total *N* = 4,554**	**HIV-positive *N* = 3,350 (73.5%)**	**HIV-negative *N* = 1,204 (26.4%)**	***p***
Male, No. (%)	3,212 (70.6)	2,486 (60.5)	728 (74.2)	<0.001^*^
Age (years), mean ± SD	46.3 ± 14.3	42.1 ± 9.3	58 ± 18.3	<0.001^**^
Stay (days), mean ± SD	22.7 ± 22.1	22 ± 20.4	24.9 ± 26	0.003^**^
APR-DRGs weight, mean ± SD	2.7 ± 2.2	2.7 ± 2	2.8 ± 2.8	<0.001^**^
Cost (euros), mean ± SD	11,620 ± 11,816	11,436 ± 9,969	12,137 ± 15,900	<0.001^**^
Re-admission, No. (%)	663 (14.6)	363 (10.8)	300 (24.9)	<0.001^*^
Death, No. (%)	761 (16.7)	454 (13.6)	307 (25.5)	<0.001^*^

*SD, standard deviation; APR-DRGs, All Patients Refined Diagnosis Related Groups*;

* *Chi-Square test*;

** * Mann-Whitney U-test*.

**Table 3:** Annual change of study variables in HIV-negative cases of *Pneumocystis* pneumonia, Spain, 2008–2012.

**Year**	***n***	**Sex (male), %**	**Age (years), mean ± SD**	**Stay (days), mean ± SD**	**APR-DRGs weight, mean ± SD**	**Cost (euros), mean ± SD**	**Re-admission, %**	**Mortality, %**
2008	201	57.2	56 ± 19.9	25.5 ± 26.1	2.5 ± 1.9	10,534 ± 7,916	26.4	24.9
2009	255	60.9	55.9 ± 19.1	23.4 ± 17.2	2.5 ± 2.1	10,598 ± 8,835	22.3	19.5
2010	193	57.8	57.2 ± 17.8	26.3 ± 27.6	2.6 ± 3.5	12,567 ± 17,288	23.4	29.7
2011	256	69.1	57.8 ± 17.6	23.7 ± 20.1	2.5 ± 3.4	12,631 ± 17,143	23.8	27.7
2012	299	56.5	61.5 ± 17.2	25.8 ± 34.1	2.7 ± 4.1	13,827 ± 21,452	28.1	26.4
Mean	240.8	60.5	57.9 ± 18.3	24.9 ± 26	2.6 ± 3.2	12,138 ± 15,900	24.9	25.5
*p*	<0.001^*^	0.022^*^	0.002^**^	0.828^***^	0.317^**^	<0.001^***^	0.527^*^	0.115^*^

*SD, standard deviation; APR-DRGs, All Patients Refined Diagnosis Related Groups (standardized stratifier in four levels of severity whose weight increases as the severity of the patient increases)*;

* *Chi-square test*;

** *ANOVA*,

*** * Kruskal Wallis H-test*.

### PcP Risk Factors

**Figure 1:**
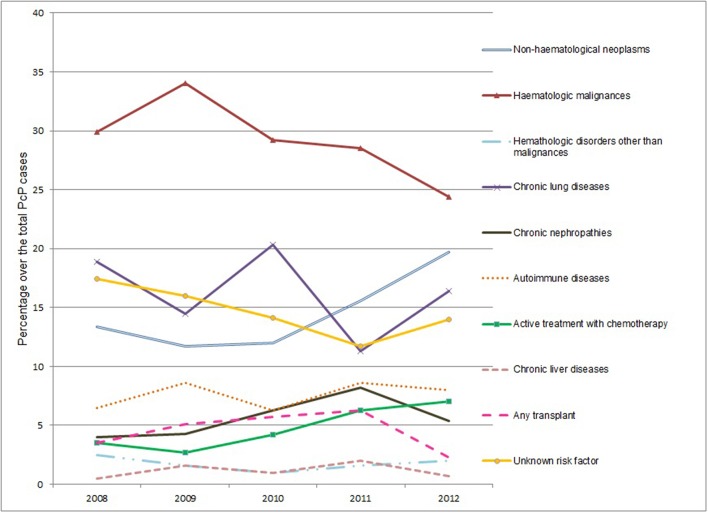
Risk factors for *Pneumocystis* pneumonia among HIV-negative patients during the study period, Spain, 2008–2012. Changes 2012 vs. 2008 (Chi-Square test): non-hematological neoplasms (*p* = 0.067); hematologic malignances (*p* = 0.177); Hematologic disorders other than malignances (*p* = 0.726); chronic lung diseases (*p* = 0.467); chronic nephropathies (*p* = 0.482); autoimmune diseases (*p* = 0.514); active treatment with chemotherapy (*p* = 0.069); chronic liver diseases (*p* = 1); any transplant (*p* = 0.307); unknown risk factor (*p* = 0.309).

A PcP risk factor was observed in 85.5% of cases, with the most common being hematological cancers (29%), chronic lung diseases (15.9%), and non-hematological neoplasms (14.9%). The other risk factors are displayed in Table 4. The annual evolution of the different risk categories during the study period are displayed in Figure 1, with changes being observed in their distribution during this period, with the most important being the increase in the proportion of patients with non-hematological neoplasms from 13.4% in 2008 to 19.7% in 2012 and in subjects who receive chemotherapy from 3.5% in 2008 to 7% in 2012 and the decrease in patients with hematological neoplasms (29.9% in 2008 and 24.4% in 2012).

**Table 4:** Risk category for *Pneumocystis* pneumonia (PcP) in HIV-negative patients, Spain, 2008–2012.

**Risk category**	**No. (%)**
Hematologic malignances	349 (29)
Chronic lung diseases	192 (15.9)
Malignances other than hematologic	179 (14.9)
Autoimmune diseases^*^	93 (7.7)
Chronic nephropathies	68 (5.6)
Treatment with chemotherapy	59 (5)
Any transplant	54 (4.5)
Hematologic disorders other than malignances	21 (1.7)
Chronic liver diseases	14 (1.2)
Unknown	176 (14.5)

* *Rheumatoid arthritis, systemic lupus erythematosus, polymyositis, dermatomyositis, chronic mixed connective tissue disease, Crohn's disease, and systemic vasculitis*.

## Discussion

The present study is the first to address nationwide the epidemiology of PcP in patients with or without HIV infection in Spain. Our results indicate that PcP currently continues to be in our country a disease with a stable incidence and that its epidemiological spectrum is changing, with a decrease in the cases of patients with HIV infection and a parallel increase in subjects without this infection being observed during the study period. Furthermore, the study confirms that PcP in patients without HIV infection has a high mortality rate and healthcare cost and it has allowed the main risk groups to be identified, among which factors previously not associated with PcP such as chronic lung diseases are found.

The potential study limitations are related to the use of administrative bases as a source of information. The CDMD is an administrative record that must be completed in all hospitals in Spain. The clinical variables are obtained through the discharge report and, as such, if the coders of the different centers interpret the clinical data recorded in a different manner, there could be reliability and variability problems.

However, the coding is carried out by experts who receive specific training and use a standardized coding regulation (17), which reduces the possibility of information bias. Moreover, the CMBD database covers 98% of hospital discharges from public hospitals in Spain (18), and a significant percentage of discharges in private hospitals, which in 2012 was more than 60% (19). Therefore, taking into account that PcP is a disease treated while the patients are hospitalized, our results are representative of the epidemiology of this disease for the Spanish territory.

In our survey, the total number of patients hospitalized because of PcP in Spain between 2008 and 2012 was 4,554, with an average annual incidence of 19.4 cases per million inhabitants that, interestingly, remained stable during the study period. The incidence observed is lower than that found in previous studies carried out in the Spanish region of Andalusia in the 1988–1999 period (34 cases per million inhabitants) (20). These findings are explained by the decrease in HIV-infected patients after the generalization of the HAART treatment and are in line with other epidemiological studies carried out in Spain (21) and Europe (4, 11, 22).

26.4% of cases of PcP were recorded in patients without HIV infection with a mean annual incidence rate in this patient group of 5.1 cases per million inhabitants, which is similar to that reported in the United Kingdom in the 2006–2010 period (5.1 cases per million inhabitants) (11), which confirms that it is not an isolated phenomenon in our country.

During the study period in this group, there was a progressive increase in the incidence of the disease and a parallel increase in the percentage of cases in this group with respect to the overall amount of patients diagnosed. This trend is also in line with previous analyses carried out by our group in which the proportion of PcP cases in patients without HIV infection was 13% in a study carried out in the Spanish region of Andalusia (1998–1999 period) (20), and of the 18% in another previous nationwide study that we carried out in Spain (2003–2007 period) (23). This trend has also been observed in the United Kingdom in the 2000–2010 period, where there was an annual increase of 9% in patients not infected by HIV, and a parallel annual decrease of 7% in patients with HIV infection (11). However, the rising PcP rate is not always true, as there was a dip from 2009 to 2010.

Regarding the epidemiological and clinical presentation spectrum, independent of the cause of immunosuppression, PcP in patients without HIV infection continue to be more severe than those observed in HIV-infected patients and they have higher intrahospital mortality and re-admission rates, as was reported in previous studies (22, 24), however, in some of them, mortality rates of up to 38% (25) have been reported. This situation could be the result of the presence of underlying diseases with a worse prognosis than HIV infection, a delay in diagnosis and the start of suitable treatment for PcP due to the lower index of suspicion (20) or else, it could be secondary to a greater pulmonary inflammatory response to *Pneumocystis* in this patient group (2, 7, 9).

During the study period, in patients without HIV infection, an increase in the mean age of patients hospitalized due to PcP was observed, results that could be related to a greater survival rate of the subjects at risk of having the disease. However, the intermediate indicators related to the complexity of the patients and the management of the PcP, such as the APR-DRG weight, mean hospitalization and the re-admission rate remained stable. Moreover, our results show that PcP episodes have a high cost, in which we observed during the period an increase in the cost of hospitalization that is not related to the greater complexity of patients or greater mean hospitalization, and which does not allow for a decrease in re-admissions and which, simply, could be in relation to the inflation that fluctuated in this period in Spain between 4.1 and 2.4% per year (13).

With regard to PcP risk factors in patients without HIV infection, those most commonly observed in our study were hematological neoplasms (29%), as occurred in previous studies (11). The proportion of cases of this subgroup is slightly lower than that which we observed in the 2003–2007 period, which was 34% (23), probably as a result of the greater use of prophylaxis, which is a now widely accepted recommendation in patients with hematological neoplasms (26, 27).

Moreover, during the study period a rise in the proportion of cases in the category of solid neoplasms was observed, increasing from 13.4% in 2008 to 19.7% in 2012, being much higher than the reported in other series (11, 16). We would have to relate these results to the increasingly more common use of aggressive chemotherapy protocols (27). Furthermore, as in other studies (11, 28), it is confirmed that patients with chronic lung diseases are currently a new risk group, being in our study the second clinical category most commonly associated with PcP. In this regard, numerous studies have highlighted that *P. jirovecii* colonization (identification of the pathogen in respiratory samples in patients which do not have pneumonia) is a common biological phenomenon in patients with chronic obstructive pulmonary disease (40.5%), cystic fibrosis (21.5%), or interstitial lung disease (33.8%) (29–31) and, as such, this group could represent an important species-specific reservoir of *Pneumocystis* infection.

Due to all of the above, PcP could currently be considered to be an emerging disease in immunocompromised subjects without HIV infection, as a result of the growing number of patients who receive immunosuppression therapy and aggressive chemotherapy protocols for the control of neoplasms, which are known risk factors for the development of PcP described above (2, 6, 8, 9, 11, 15, 16) and for the emergence of new and unknown risk groups such as patients with chronic lung diseases in which there is a delay in diagnosis and there are no defined chemoprophylaxis guidelines.

## Conclusion

The results show that, despite the general belief that PcP is an uncommon disease after the generalization of HAART for HIV infection, its epidemiological impact is still significant in Spain. Its incidence has increased in patients without HIV infection in whom, in addition to classic risk factors such as solid or hematological neoplasms, new emerging risk groups have been identified, such as patients with chronic lung diseases; however, in almost 15% of them it was not possible to identify the predisposing factor, which is not surprising, bearing in mind that 10% of the general population may be colonized by the disease (32). PcP mortality in patients without HIV infection continues to be high (25.5%), and an increase in the mean age and in the cost of caring for patients was observed during the study period, not related to the increase of the clinical complexity or mean hospitalization.

Our findings justify the need to carry out new studies that allow for a better characterization of PcP risk groups in patients without HIV infection and, in this manner, define more effective prevention and early diagnosis guidelines that allow the growth and mortality of this devastating illness to be halted.

## Data Availability Statement

The datasets generated for this study are available on request to the corresponding author.

## Ethics Statement

The project was approved by the Clinical Research Ethics Committee (CEIC) of the Hospital Universitario Virgen del Rocío. According to Spanish law, patient consent is not required for register-based studies. No further ethical permissions are required for the analyses of these anonymized patient-level data.

## Author Contributions

EC and FM conceived and designed the research. EP-D and FM collected and analyzed the data and wrote the draft of the manuscript. JG performed the statistical analysis. EP-D, FM-V, CH, and FM contributed to the development of the study and interpreted the data. All authors reviewed and approved the final version of the manuscript.

### Conflict of Interest

The authors declare that the research was conducted in the absence of any commercial or financial relationships that could be construed as a potential conflict of interest.
